# Gender Differences in the Wechsler Intelligence Scale for Children in a Large Group of Italian Children with Attention Deficit Hyperactivity Disorder

**DOI:** 10.3390/jintelligence11090178

**Published:** 2023-09-05

**Authors:** David Giofrè, Enrico Toffalini, Lorenzo Esposito, Cesare Cornoldi

**Affiliations:** 1 DISFOR, University of Genoa, 16128 Genova, Italy; 2 Department of General Psychology, University of Padua, 35131 Padova, Italy; 3 Department of Developmental and Social Psychology, University of Padua, 35131 Padova, Italy

**Keywords:** intelligence, attention deficit hyperactivity disorder, ADHD, Wechsler intelligence scale for children, WISC, coding

## Abstract

Despite being repeatedly investigated in children with typical development, research on gender differences in intellectual abilities in specific groups of children, including children with attention deficit hyperactivity disorder (ADHD), has been scarce. In this paper, we evaluated the performance of a large group of Italian children with ADHD using the WISC-IV. We aimed at investigating the presence of gender differences using a multi-group confirmatory factor analysis approach. Results showed that the WISC is largely gender-invariant. However, some tasks present non-invariant patterns (block design and coding). Differences at the latent level also showed some differences (favoring boys) in the verbal comprehension index. Conversely, differences at the latent level were not found in the full-scale IQ or in the other main indices. These results have theoretical and practical implications.

## 1. Introduction

The existence of gender differences in cognitive abilities has been harshly debated in the current literature. Recently, it has become clear that some gender differences exist in certain specific abilities (Geary 2021). Several different theories have been proposed, but the most influential maintain that both social and biological factors might be related to differences in some domains (Miller and Halpern 2014). Differences in specific aspects have repeatedly been observed, for example, with girls outperforming boys in tasks requiring attentional control to a larger extent and boys outperforming girls in some tasks requiring the visuospatial manipulation of the stimuli (Geary et al. 2021). These findings have been replicated using several instruments, including, for example, the Wechsler intelligence scale for children (WISC) (Giofrè et al. 2022a). These differences occur not only in children with typical development but also in children with various neurodevelopmental disabilities, including, but not limited to, children with specific learning disabilities (Giofrè et al. 2022b). While investigated in some groups, gender differences were less investigated in other groups, for example, in children with attention deficit hyperactivity disorder (ADHD), where the gender seems crucial, at least from an epidemiological point of view; the probability of having ADHD is four times higher in boys than in girls (American Psychiatric Association 2013). It is also worth noting that the cognitive profile of children with ADHD usually presents with some peculiarities; in fact, children with ADHD are particularly impaired in tasks such as coding that require attentional resources to a larger extent (Gomez et al. 2016; Jacobson et al. 2011; Mayes and Calhoun 2006). The aim of the current report is to shed light on this by investigating gender differences, if any, using the WISC-IV (Wechsler 2003). In fact, in Italy, the WISC-IV is the most used battery as some of the most widely used batteries (e.g., the WISC-V, the Woodcock–Johnson, and the Stanford–Binet) have not been validated in Italian, making it very hard to use other batteries for the intellectual assessment of children with typical and atypical development (Cornoldi 2020). 

Several batteries are employed to assess the intellectual profile of children, but the most used one in some countries remains the WISC-IV (Evers et al. 2012). In Italy, virtually all children are assessed with the WISC-IV; this is also because the WISC-V (Wechsler 2014), which was recently standardized in several countries, has not yet been adapted in Italian. The WISC-IV consists of several indices, such as verbal comprehension (VCI), perceptual reasoning (PRI), working memory (WMI), processing speed (PSI), and the full-scale IQ (FSIQ). Indices are tapped by ten principal subtests, which are supposed to measure their underlying abilities. 

Before addressing gender differences in children with atypical development, it is useful to describe the current *status quo* for children with typical development. Giofrè et al. (2022a) recently performed a meta-analysis on gender-related differences using the WISC. Results showed that differences were trivial in the overall cognitive performance (i.e., FSIQ), but the authors found that girls performed better in some tasks, for example, those requiring a rapid speed of processing and the maintenance of attention on the task for a short period of time (e.g., coding), while boys performed better on other tasks, for example, those requiring the visual manipulation of the stimuli (e.g., block design). It is not clear, however, whether this pattern of results will hold true in children with ADHD. In general, meta-analyses like the one carried out by Giofrè et al. (2022a) are particularly important, but they typically fail to detect if a test presents some issues, for example, it is not possible to estimate differences at the latent level or to control for measurement invariance. In fact, in many circumstances, it would be more appropriate to use measurement invariance using, for example, a multi-group confirmatory factor analysis (MGCFA) approach (Hunt and Carlson 2007). In fact, these results are not limited to the WISC, and recent evidence using other intelligence scales (e.g., WAIS, DAS, and Woodcock–Johnson) confirms that girls present with some advantages in processing speed tasks, while boys present with advantages in tasks requiring the mental manipulation of the stimuli (Pezzuti et al. 2020; Keith et al. 2011, 2008). 

Measurement invariance, performed via MGCFA, is a statistical method that allows estimating whether a test is measuring the same construct across different groups (Meredith 1993). Under measurement invariance, parameters are supposed to be fully invariant across groups (Meredith 1993). Several different steps can be taken to measure measurement invariance: configural, which is testing the assumption that the same factorial structure is tenable; metric, which is testing if loadings are invariant; total scalar, which is testing the invariance of intercepts; partial scalar, which is allowing some intercepts to be not invariant; residuals variances, which is testing the invariance of residuals; latent variances, which is testing the invariance of latent variables; and finally, latent means, which is assessing whether means, at the latent level, are the same or differ across groups. From a theoretical point of view, it is interesting to see whether some parameters are non-invariant across groups; for example, latent mean invariance can be used to understand whether mean differences between groups on some indicators are genuinely reflected by latent group differences in the latent factors (Meredith 1993). This approach has been used for testing differences across different groups of children, for example, with typical or atypical development (Giofrè and Cornoldi 2015); or between boys and girls, using the WISC-IV (Pezzuti and Orsini 2016; Goldbeck et al. 2010; Li et al. 2016). However, the research area of gender differences in the WISC in children with ADHD has not been fully investigated. 

Children with ADHD show a very typical profile in the WISC as they are very often characterized by lower performance in tasks measuring processing speed and working memory, while at the same time having relatively higher performance on other tasks (Mayes and Calhoun 2006; Cornoldi et al. 2023; Thaler et al. 2013). However, research on gender differences in the WISC in children with ADHD is less abundant. Notably, research in this area is typically drawn from relatively small samples and does not use more sophisticated analyses such as MGCFA, which presents with several advantages: (i) one can formulate and test a variety of competing hypotheses; (ii) decisions made using MGCFA are based on fit indices; and (iii) the use of goodness-of-fit measures and the explicit comparison of competing models provide a more transparent justification for the acceptance or rejection of the hypothesis (for an extensive discussion, see Dolan 2000).

To the best of our knowledge, gender differences in the WISC in children with ADHD, using MGCFA, have not yet been tested. Therefore, some crucial questions related to gender differences in the intellectual functioning of children with ADHD have not yet received a clear response. For example, Cornoldi et al. (2023) found that the proportion of children with ADHD who were intellectually gifted was higher in boys than in girls. Furthermore, on the opposite side, we do not know the extent to which the specific gender differences found in the typical population are also present in children with ADHD.

The main aim of this study is to shed light on gender differences in the intellectual profile of children with ADHD using the WISC-IV. To achieve this, an MGCFA was performed, testing several alternative models. Once the full (or partial) factorial invariance was established, we also aimed at looking at differences in indices and subtests. 

## 2. Materials and Methods

### 2.1. Sample

The sample consisted of children diagnosed with ADHD according to the ICD-10 coding system (F90.0; N = 1051). Participants with incomplete personal information or with a full-scale intelligence quotient (FSIQ) lower than 70, which indicates the presence of an intellectual disability, were excluded from the analyses. Our final sample consisted of 942 participants: 763 boys (M_age_ = 10.28 years, SD = 2.53) and 179 girls (M_age_ = 10.15, SD = 2.69), or about 4 boys for each girl diagnosed with ADHD, which is consistent with epidemiological data (American Psychiatric Association 2013; Cantwell 1996). The two groups did not differ statistically in age, *t*(256.66) = 0.62, *p* = .54, Cohen’s *d* = 0.05, 95% *CIs* [−0.11, 0.22]. It is worth noting that a smaller sample of girls might affect precision of the estimates in this group: standard errors were about twice or three times larger in girls than they were in boys. In fact, there are no strict rules for sample sizes in SEMs, but at least 5–10 statistical units per estimated parameters are recommended as a rule of thumb (Bollen 1989). The model presented below with most free parameters was the unconstrained multi-group higher-order model, with 34 parameters estimated in each group. This corresponded to 22.4 observations per parameter in boys and 5.3 observations per parameter in girls.

### 2.2. Instrument

The Italian version of the WISC-IV was used (Orsini et al. 2012). The present study focused on the standardized scores on the 10 core subtests that are routinely administered to all children during the clinical assessment. These subtests are grouped by four indices: the verbal comprehension index (VCI), which is measured by vocabulary (VC; name objects or explain the meaning of words), similarities (SI; explain what is similar between two objects of concepts), and comprehension (CO; explain common-sense social and practical knowledge). The perceptual reasoning index (PRI), which is measured by block design (BD; manipulate colored plastic cubes to reproduce a visual pattern), picture concepts (PCn; categorize objects that share a characteristic in common), and matrix reasoning (MR; complete a visual pattern, measures fluid reasoning). The working memory index (WMI), which is measured by digit span (DS; listen to and repeat a series of digits presented orally), and letter–number sequencing (LN; listen to, order, and repeat a mixed series of digits and numbers presented orally). The processing speed index (PSI), which is measured by coding (CD; quickly draw symbols associated with other symbols in a booklet) and symbol search (SS; quickly determine whether a target symbol appears in a search group, on a booklet). All subtests, except those in PSI, present items of increasing difficulty and the administration is discontinued when the child fails a certain number of items in a row. In addition to the four main indices, a full-scale IQ (FSIQ) is calculated by combining all core subtests. In the present study, only the 10 core subtests were used.

### 2.3. Data Analysis

**Figure 1:**
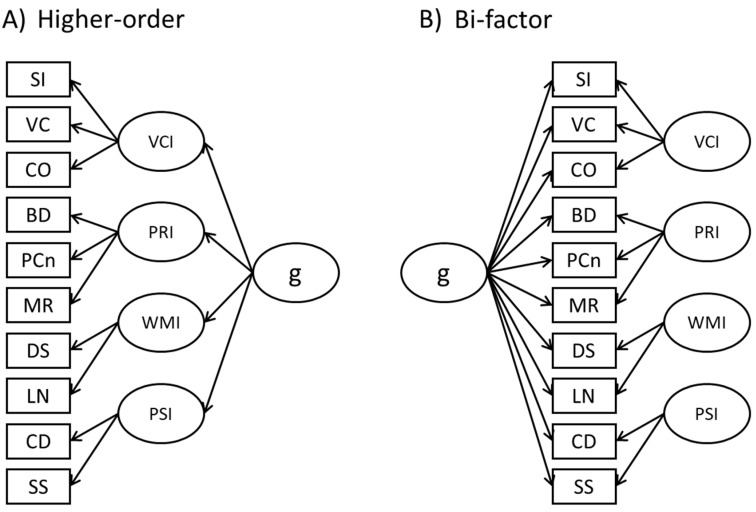
Configurations of the models tested. (**A**) represents the higher-order model, while (**B**) represent the bi-factor model. Note. VC: vocabulary; CO: comprehension; BD: block design; PCn: picture concepts; MR: matrix reasoning; DS: digit span; LN: letter–number sequencing; CD: coding; SS: symbol search; VCI: verbal comprehension index; PRI: perceptual reasoning index; WMI: working memory index; PSI = processing speed index; FSIQ: full-scale intelligence quotient.

Analyses were performed with R (R Core Team 2023). The lavaan package (Rosseel 2012) was used for model fitting. Our analytic strategy involved two steps. First, CFAs were performed to evaluate the factor structure and to choose the most suitable model. Then, the selected model was compared using MGCFA to check for measurement invariance between boys and girls. To outline the factor structure of the WISC-IV, we tested the traditional higher-order model, with four latent factors, i.e., verbal comprehension (VCI), perceptual reasoning (PRI), working memory (WMI), and processing speed (PSI), which were all loading on the higher-order general factor (g). Secondly, we tested a bi-factor model in which subtests loaded simultaneously on their respective latent variables and, at the same time, on the general factor. This approach has already been used with the WISC and using Italian participants (see Kush and Canivez 2021 for a recent discussion). Tested models are shown in Figure 1. We only had some missing data (<1%) and we therefore opted for listwise deletion to simplify the analyses. Maximum likelihood (ML) was used in all the analyses.

Different goodness-of-fit statistics were computed to evaluate model fit. In particular, we considered the chi-square (*χ*^2^), the root mean square error of approximation (*RMSEA*), the standardized root mean square residual (*SRMR*), the comparative fit index (*CFI*), the non-normed fit index (NNFI), the Akaike information criterion (*AIC*), and the Bayesian information criterion (*BIC*). Cut-off values for fit were considered good if chi-square (*χ*^2^) significance was present, *CFI* and NNFI were greater than 0.95, *RMSEA* was lower than 0.06, and the *SRMR* was lower than 0.05 (Hu and Bentler 1999). The most plausible model was selected based on goodness-of-fit criteria and by considering the relative difference in indices *AIC* and *BIC* between the competitive models.

After model selection, we explored measurement invariance across boys and girls using MGCFA. To evaluate invariance between boys and girls, we considered the general guidelines proposed by Chen (2007). In particular, a decrease in *CFI* of less than 0.01 (Δ*CFI*), an increase in *RMSEA* of less than 0.015 (Δ*RMSEA*) between models, and acceptable model fit indices are claiming for model invariance (Cheung and Rensvold 2002). Similarly, a decrease in NNFI of less than 0.01 (ΔNNFI) and an increase in *SRMR* of less than 0.015 (Δ*SRMR*) were considered acceptable for invariance. Absence of chi-square significance difference (Δ*χ*^2^) was also used for model invariance. Tests of measurement invariance followed the conventional series of steps. Initially, we tested configural invariance, in which all model parameters were free to vary across groups. If the fit indices were acceptable, the model configuration was regarded as adequate for both groups and configural invariance was established. Second, metric invariance was assessed by constraining factor loadings to equality in the two groups. If this model did not substantially lose fit as compared to the previous one, metric invariance was established. In the third step, scalar invariance was tested by constraining intercepts to equality across groups. Fourth, invariance of residuals was tested by constraining the variance associated to the observed residuals to equality between groups. Once these steps were completed, strict invariance was established, which implies that the two groups can be directly compared on their latent variable scores. The subsequent steps involved testing the equality of variances and means of the latent factors. Once again, this was tested by constraining the parameters to equality between groups and testing whether there was a substantial loss of fit via model comparisons with the models fitted in the previous steps. When model comparisons suggested lack of invariance, we tested partial invariance by constraining some, but not all, parameters to equality across groups. We followed an iterative procedure by freeing a pair of parameters at a time, starting from those that had the largest influence on the model fit, as indicated by the modification indices.

## 3. Results

### 3.1. Descriptive Statistics

Descriptive statistics and Cohen’s *d* for each subtest and index are provided in Table 1 and Table 2, respectively. Table 2 suggests that scores in the verbal reasoning index and in the perceptual reasoning index are relatively higher compared to scores in the working memory and the processing speed indices: averaged VCI–PRI scores were higher than WMI–PSI scores by about one standard deviation; Cohen’s *d* = 1.34 [1.24, 1.44]. Gender differences in observed scores, albeit modest in terms of magnitude, seem to emerge on some specific subtests (i.e., block design and coding, with |Cohen’s *d*s| > 0.30, see Table 1). However, results at the observed level can be different from those at the latent level. It is also worth mentioning that indices presented in Table 2 are calculated from manifest variables and are not based on factor analyses. For all these reasons, it is more appropriate to use a latent variable approach. 

### 3.2. Model Selection

CFAs were performed to test different theoretical models. Both models presented good fit indices (Table 3). Between higher-order and bi-factor models, the latter had slightly better fit indices, but it also presented a larger *BIC*. In addition, the bi-factor model required constraining some loadings to equality across subtests (for both working memory and processing speed factors) in order to be identified. Furthermore, in the first step of measurement invariance, it failed to compute standard errors, making the credibility of estimates uncertain. Therefore, we chose to perform MGCFA using the higher-order model to test for measurement invariance, which showed no estimation problems.

### 3.3. Invariance across Boys and Girls

An MGCFA was fitted with the higher-order model and with two CFAs, one for boys and one for girls, respectively. Results showed good fit indices in all cases (see Table 4). The *χ*^2^ reaches statistical significance for males, but not for females, which can reflect the difference in the sample size (Kelloway 1995).

To test invariance across the two gender groups, an MGCFA on the higher-order solution was performed following sequential steps. Firstly, configural invariance of the higher-order model was established (see Table 4). Since configural invariance was satisfied, in the following step, we constrained the loadings to equality across the two groups. The chi-square difference was not significant, and Δ*CFI* and Δ*NNFI* were less than 0.01, Δ*RMSEA* and Δ*SRMR* were less than 0.015, and both *AIC* and *BIC* indices decreased. Therefore, metric invariance was established. The subsequent step, however, showed lack of total scalar invariance. In fact, through constraining subtests’ intercepts, the fit worsened markedly. Particularly, the chi-square difference test was not significant, however, Δ*CFI* and Δ*NNFI* were 0.011 and 0.01, respectively; *AIC* index increased, whereas *BIC* decreased. Thus, we rejected this model. Subsequently, we ran a series of models to test partial scalar invariance by freeing one subtest intercept at a time. Results suggested that intercepts for block design and coding needed to be released and free to vary between boys and girls. The resulting model with partial scalar invariance showed no substantial loss of fit, and the chi-square difference was not significant and presented lower *AIC* and *BIC* (Table 5). 

**Figure 2:**
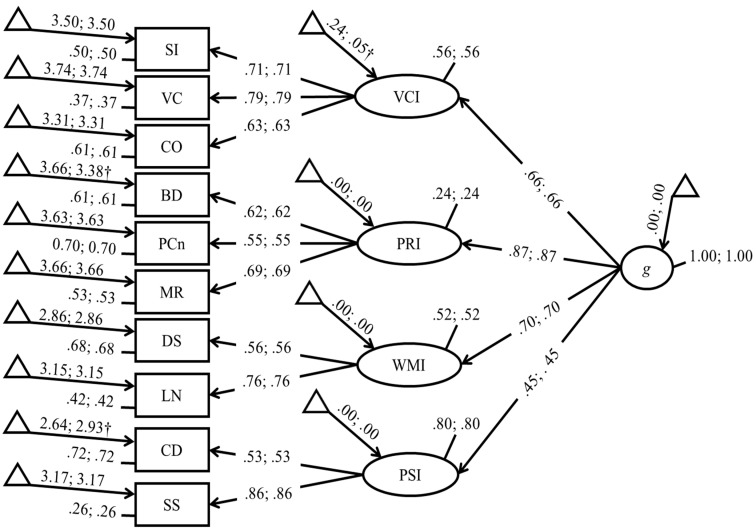
Standardized coefficients of the final model with free latent intercepts for VCI. The left coefficient is for males, and the right coefficient is for females. All parameters, except those with a †, are constrained to equality between groups based on measurement invariance assessment. Note. VC: vocabulary; CO: comprehension; BD: block design; PCn: picture concepts; MR: matrix reasoning; DS: digit span; LN: letter–number sequencing; CD: coding; SS: symbol search; VCI: verbal comprehension index; PRI: perceptual reasoning index; WMI: working memory index; PSI = processing speed index; FSIQ: full-scale intelligence quotient.

As partial scalar invariance was established, we proceeded to test residuals invariance and then invariance of latent variances and latent means by constraining these last sets of parameters to equality across groups. Even for these last steps, results showed good fit indices, no significance of chi-square differences, and progressively lower *AICs* and *BICs*. Therefore, the final model implied that the latent means could be set to equality across boys and girls with ADHD. Nonetheless, analysis of the model with latent means still free to vary across groups suggested that some minor between-group differences might not be excluded. Specifically, a significant difference in the latent mean of VCI (favoring boys) emerged; *β* = −0.19, 95% *CIs* [−0.36, −0.02], *p* = .029. A comparison between the final model (i.e., the one with all latent means constrained to equality across groups) and an alternative model, in which only the latent mean of VCI left free to vary, presented only some evidence in favor of this model; Δ*χ*^2^(2) = 4.77, *p* = 0.09, Δ*AIC* = −0.77, Δ*BIC* = +8.86. The standardized coefficients of the final latent means model, but with free latent mean for VCI, are shown in Figure 2.

## 4. Discussion

The aim of the current report was to investigate the presence of gender differences in the WISC-IV in a large sample of children with ADHD. Results confirmed the presence of partial invariance, in which intercepts of block design and coding were non-invariant between the two groups. We also found some evidence for the presence of a latent difference in one of the main indices (i.e., verbal comprehension).

Results of the MGCFA are very interesting. For a start, the fact that block design and coding presented non-invariant intercepts is in line with the general literature on children with or without typical development. A recent meta-analysis on the WISC-IV in children with typical development shows that block design and coding are probably the tasks in which differences in favor of boys (block design) and of girls (coding) are largest in terms of magnitude (Giofrè et al. 2022a). This result also seems to be very stable in children with various neurodevelopmental disabilities, including children with specific learning disabilities (Giofrè et al. 2022b). The presence of non-variant intercepts generally reflects the fact that the differences in these subtests largely exceed what is expected based on the latent differences in their corresponding latent factors. Reasons behind these differences are probably related to the relative advantages of boys in some tasks requiring the visual manipulation of the stimuli (Geary 1995, 2022; Halpern and Wai 2019); girls generally outperform boys in processing speed tasks, probably due to their faster processing in writing speed and associated learning and their faster retrieval from secondary memory (Halpern and Wai 2019; Giofrè et al. 2022a; Keith et al. 2011).

Looking at the results from the MGCFA latent level, our results are generally in accordance with the literature. We did not find any evidence of differences between boys and girls in the g-factor at the latent level or in the FSIQ at the manifest level, which is in line with results in children with ADHD, in which differences are scattered, not statistically significant, and small in terms of magnitude (Seymour et al. 2016; Wang et al. 2019; Yang et al. 2004; Lambek et al. 2010; O’Brien et al. 2010; Muñoz-Suazo et al. 2019). Comparable results from the MGCFA are obtained in the PRI and WMI indices, in which differences seem to be scattered and not always statistically significant (Seymour et al. 2016; Wang et al. 2019; Yang et al. 2004; O’Brien et al. 2010; Muñoz-Suazo et al. 2019; Tang et al. 2019). However, the results at the observed level are somehow different in the PRI, but only after considering the non-invariance of block design. Conversely, results on the PSI at the manifest level seem to be consistent, with virtually all studies showing an advantage for girls in this index (Seymour et al. 2016; Wang et al. 2019; Yang et al. 2004; O’Brien et al. 2010; Muñoz-Suazo et al. 2019; Tang et al. 2019). It is worth noting, however, that the PSI is calculated based on only two subtests, and results can be largely influenced by small variation in one subtest (e.g., coding). This is probably why the results using the MGCFA show that the latent mean of this factor is similar between the two groups, but only after having considered the non-invariance of the coding task. Finally, differences in the VCI seem to be more elusive, and most of the studies, albeit not every study, show some advantage for boys (Seymour et al. 2016; Wang et al. 2019; O’Brien et al. 2010; Muñoz-Suazo et al. 2019; Tang et al. 2019). The limits of the aforementioned literature, however, are the sample size, which was quite small in most of the cases, and the use of traditional statistical approaches (rather than an MGCFA), which makes it hard to draw reliable conclusions. In fact, when looking at the results from the MGCFA, we did find strong evidence of a difference between the latent means of boys and girls in the VCI.

In the current report, we found larger differences in two indices, that is, PRI favoring boys and PSI favoring girls. However, this was only at the observed level, and in fact, latent means in these two indices were not statistically different from each other. This finding is probably a consequence of the fact that block design, measuring PRI, and coding, measuring PSI, are affecting these two indices. In fact, it is very likely that the exclusion of these two tasks from these indices would probably determine a sensible reduction in the standardized difference in these two indices. The non-invariance in the intercepts, in fact, seems to indicate that there are not genuine differences in the latent factors, but only in some tasks (i.e., block design and coding). As for VCI, the results at the latent level are remarkably interesting. We noticed that intercepts of tasks measuring the VCI factor are invariant, but some differences appear at the latent level, which might indicate a genuine difference in this latent factor. This is not surprising, since we noticed that a similar finding was obtained in children with both typical and atypical development (Giofrè et al. 2022a, 2022b). However, the results should be taken with caution since the evidence for the presence of a latent mean difference on this factor was not particularly strong. 

The results of the current report should be replicated in future studies. One limitation of our paper is that although the sample size is appropriate, the number of girls is not particularly large. This reflects the fact that the prevalence of ADHD is higher in boys (American Psychiatric Association 2013). It would also be interesting to compare children with ADHD using the newly released WISC-V, which is unfortunately unavailable in several countries (including, for example, Italy). It is also worth noting that our analyses are limited to the main subtests. In fact, supplementary subtests are also not routinely used in clinical practice because they do not typically concur with the calculation of the IQ (Flanagan and Kaufman 2004). However, we believe that additional subtests can be extremely useful and important in the context of the MGCFA. In fact, only having two subtests, as in the case of the WMI and the PSI, makes it hard to evaluate the latent means of these indices, making it necessary to replicate these findings using the entire scale rather than focusing only on the main subtests. 

## 5. Conclusions

Despite these limitations, we believe that this study, performed on a large sample of children with ADHD and using a sophisticated statistical approach (MGCFA), can make an important contribution to the field.

## Figures and Tables

**Table 1:** Descriptive statistics of WISC-IV subtests’ scores by group.

Subtest	Group	M	SD	Cohen’s *d*	95% CIs
SI	M	10.53	2.98	0.124	[−0.039, 0.287]
	F	10.17	2.69		
VC	M	10.61	2.73	0.207	[0.044, 0.370]
	F	10.05	2.64		
CO	M	10.76	3.18	0.040	[−0.123, 0.203]
	F	10.64	3.04		
BD	M	10.71	2.9	0.309	[0.145, 0.472]
	F	9.79	3.25		
PCn	M	11.01	3.03	0.048	[−0.115, 0.211]
	F	10.87	3.11		
MR	M	10.70	2.86	0.166	[0.002, 0.329]
	F	10.22	3.09		
DS	M	7.51	2.63	0.027	[−0.136, 0.190]
	F	7.44	2.56		
LN	M	8.15	2.56	0.001	[−0.165, 0.167]
	F	8.15	2.65		
CD	M	7.51	2.98	−0.315	[−0.478, −0.151]
	F	8.44	2.74		
SS	M	8.78	2.90	−0.044	[−0.207, 0.119]
	F	8.91	2.78		

Note. SI: similarities; VC: vocabulary; CO: comprehension; BD: block design; PCn: picture concepts; MR: matrix reasoning; DS: digit span; LN: letter–number sequencing; CD: coding; SS: symbol search. M = males (boys); F = females (girls).

**Table 2:** Descriptive statistics of WISC-IV index scores by group.

Index	Group	M	SD	Cohen’s *d*	95% CIs
VCI	M	103.86	14.69	0.145	[−0.018, 0.308]
	F	101.75	13.69		
PRI	M	105.06	14.48	0.219	[0.055, 0.382]
	F	101.83	15.9		
WMI	M	87.08	13.02	0.038	[−0.125, 0.201]
	F	86.58	13.69		
PSI	M	88.96	14.84	−0.205	[−0.368, −0.042]
	F	91.95	13.42		
FSIQ	M	96.82	13.31	0.094	[−0.069, 0.257]
	F	95.58	12.97		

Note. VCI: verbal comprehension index; PRI: perceptual reasoning index; WMI: working memory index; PSI = processing speed index; FSIQ: full-scale intelligence quotient. M = males (boys); F = females (girls).

**Table 3:** Fit indices of the confirmatory factorial models of the WISC-IV.

Model	*χ* ^2^	*p*	*χ*^2^/*df*	*RMSEA*	*SRMR*	*CFI*	*NNFI*	*AIC*	*BIC*
Model 1	86.578	<.001	2.79	0.044	0.040	0.971	0.958	43,358	43,473
Model 2	47.777	.001	2.28	0.037	0.028	0.986	0.970	43,339	43,503

Note. Model 1 = higher-order with g on top; Model 2 = bi-factor.

**Table 4:** Fit indices of the multi-group confirmatory factorial higher-order model and the two confirmatory factorial higher-order models for the boys and girls groups, fitted separately.

Group	*χ* ^2^	*p*	*χ*^2^/*df*	*RMSEA*	*SRMR*	*CFI*	*NNFI*	*AIC*	*BIC*
M	86.388	<.001	2.79	0.049	0.043	0.965	0.950	35,231	35,341
F	32.627	.387	1.05	0.018	0.047	0.995	0.993	8100	8176
MG	119.015	<.001	1.92	0.045	0.040	0.970	0.957	43,371	43,698

Note. MG = multi-group comparison; M = males (boys); F = females (girls).

**Table 5:** Model fit for the higher-order model tested for invariance across boys and girls groups.

*Step of Invariance*	*χ* ^2^	*p*	*χ*^2^/*df*	*RMSEA*	*SRMR*	*CFI*	*NNFI*	*AIC*	*BIC*	Δ*χ*^2^	*p* (Δ*χ*^2^)	Δ*RMSEA*	Δ*SRMR*	Δ*CFI*	Δ*NNFI*
Configural	119.02	<.001	1.92	0.045	0.040	0.970	0.957	43,371	43,698						
Metric	131.69	<.001	1.85	0.043	0.046	0.969	0.960	43,366	43,650	12.675	0.177	−0.002	0.005	−0.002	0.003
Total Scalar	157.17	<.001	2.07	0.048	0.048	0.958	0.950	43,381	43,641	25.477	0.178	0.005	0.003	−0.011	−0.010
Partial Scalar	136.06	<.001	1.84	0.043	0.046	0.968	0.961	43,364	43,634	4.371	0.224	0.000	0.000	−0.001	0.001
Residuals Variances	147.80	<.001	1.76	0.041	0.047	0.967	0.965	43,356	43,577	11.738	0.303	−0.002	0.001	−0.001	0.004
Latent Variances	155.49	<.001	1.75	0.040	0.050	0.966	0.965	43,353	43,551	7.695	0.174	0.000	0.003	−0.001	0.001
Latent Means	163.69	<.001	1.78	0.040	0.052	0.964	0.965	43,352	43,525	8.199	0.146	0.000	0.001	−0.002	0.000

## Data Availability

Data are unavailable due to privacy.
